# Anticancer Potential of Silibinin Loaded Polymeric Nanoparticles against Breast Cancer Cells: Insight into the Apoptotic Genes Targets

**DOI:** 10.31557/APJCP.2021.22.8.2587

**Published:** 2021-08

**Authors:** Ali Pourgholi, Mehdi Dadashpour, Akram Mousapour, Akram Firouzi Amandi, Nosratollah Zarghami

**Affiliations:** 1 *Department of Pharmaceutical Chemistry, Faculty of Pharmacy, Eastern Mediterranean University, Famagusta, TR North Cyprus, Turkey. *; 2 *Stem Cell Research Center, Tabriz University of Medical Sciences, Tabriz, Iran. *; 3 *Department of Biotechnology, School of Medicine, Semnan University of Medical Sciences, Semnan, Iran. *; 4 *Department of Immunology, School of Medicine, Tabriz University of Medical Sciences, Tabriz, Iran. *; 5 *Department of Clinical Biochemistry and Laboratory Sciences, Faculty of Medicine, Tabriz*, *Iran. *; 6 *Department of medicine, Faculty of medicine, Istanbul Aydin University, Istanbul, Turkey. *; 7 *Drug Applied Research Center, Tabriz University of Medical Sciences, Tabriz, Iran.*

**Keywords:** Silibinin, PLGA, PEG, polymeric nanoparticles, apoptosis, breast cancer

## Abstract

Silibinin (SIL) is a natural polyphenolic flavonoid with multiple biological and anti-cancer features. However, the complex hydrophobic nature and inadequate bioavailability of SIL hinder its efficiency at tumor sites. Investigating the possibility of an extensive strategy for better treatment of breast cancer, we carried out a comparative exploration of the inhibitory effect of SIL and SIL loaded PLGA-PEG nanoparticle (SIL-NPs) on the expression of the proapoptotic target genes, which is considered as an influential molecular target for treatment of breast cancer. The main diameter of SIL-NPs was 220 ± 6.37 and 150 ± 23.14 nm via DLS and FE-SEM respectively. Furthermore, the zeta potential of PLGA-PEG and SIL-NPs was -5.48±0.13 and -6.8±0.26 mV respectively. SIL encapsulation efficiency and drug release were determined by about 82.32 % by analyzing the calibration curve of SIL absorbance at 570 nm. Cytotoxicity of SIL and SIL-NPs was conducted by MTT assay after 24, 48, and 72 h of exposure times, and the gene expression levels of apoptotic genes, *p53* and *hTERT* was measured by real-time PCR. Evaluation of drug toxicity revealed that SIL-NPs represents higher cytotoxic effects than pure SIL in a time and dose-dependent manner. Moreover, the results demonstrated that SIL-NPs could induce apoptosis in breast cancer cells by upregulation of* caspase-3, caspase-7, p53 *and* Bax*, along with* Bcl-2, hTERT, survivin* and *Cyclin D1* down regulation. Our results indicated that *PLGA-PEG *can be used as stable carriers in nano-dimensions and SIL-NPs can be considered as a promising pharmacological agent for cancer therapy.

## Introduction

With the increasing number of cancer-related death worldwide, cancer has become one of the most important health issues and second principal causes of mortality estimated to reach for 606,520 people deaths in the United States in 2020 (national cancer institute) (Nejati-Koshki et al., 2014; Farajzadeh et al., 2018; Pahlavan et al., 2020). Among three prevalent neoplastic disease in women including colorectal cancer, lung cancer, breast cancer accounts for about 30% of all diagnosed cases and impacts over 1.5 million women each year (Lotfi-Attari et al., 2017; Jeddi et al., 2019; Nikmanesh et al., 2020). Several established risk factors have been reported for breast cancer such as sex, aging, estrogen, family history, gene mutations and unhealthy lifestyle (Maasomi et al., 2017; Abbasi et al., 2021; Nejati et al., 2021a). In addition, low-risk single nucleotide polymorphisms, mutations of high-or moderate-risk genes e.g., BRCA1, BRCA2, TP53, ATM and CHEK are common factors contributing breast cancer (Cancer, 2019; Du et al., 2019). Indeed, breast cancer is an outcome of changes in cellular processes that promote cell proliferation and metastasis along with suppressing apoptosis (Javan et al., 2019; Pahlavan et al., 2020; Rasouli et al., 2020). 

Estrogen receptor (ER) is predominantly overexpressed invasive breast tumors representing an important hallmark for the prophecy and forecast of breast cancer. Numerous variables in tissue processing can influence the ER expression level in breast tumors (Caruana et al., 2020).

The presence of ER in mammalian tumors increases hormone therapy responses by 55% to 80%. In addition, ER has been reported to be contributed with the cytotoxic effects of flavonoids on breast cancer cells (Wang and Yuan, 2018).

The most common approaches that used today to treatment of cancer include surgery, chemotherapy, radiotherapy and their combination. However, these approaches encounter several hindrances and side effects such as systemic toxicity, drug resistance, high reoccurrence, and various side effects including neutropenia, thrombocytopenia, anemia, nausea, and hair loss (Mohammadian et al., 2017; Mellatyar et al., 2018). In this regard, searching for novel therapeutic agents with natural sources passing higher acceptability and safety along with limited side effects have been greatly taken into the attention (Rasouli and Zarghami, 2018; Faramarzi et al., 2019). Besides, an effective combination of natural agents with currently used chemotherapeutics could result in a synergic inhibitory effect and higher therapeutic effects (Chatran et al., 2018).

Flavonoids are a group of plant derived polyphenolic substances with anti-oxidant possessions that can be exploited in cancer therapy (Tavsan and Kayali, 2019). 

SIL (C_25_H_22_O_10_) is flavonoid extracted from silybum marianum flavinoligan (known as milk thistle), with natural anti-inflammatory properties inhibiting oxidative stress (Dadashpour et al., 2017; Chen et al., 2020). Several studies have indicated the anti-proliferative effect of SIL as well as G1 arrest property, apoptosis induction, and decreasing hTERT expression (Amirsaadat et al., 2010; Abdollahi et al., 2015). Hence, the combination of SIL with current chemotherapeutic agents could enhance the efficiency of chemotherapy both in vivo and in vitro (Gohulkumar et al., 2014). However, the hydrophobic and multiring structure of SIL, as well as limited bioavailability, results in low aqueous solubility of SIL hindering its preclinical and clinical oral administration (Gogoi et al., 2018). In this regard, several approaches have been employed to enhance SIL bioavailability including complexation with soluble derivatives e.g., cyclodextrins, phosphatidylcholine, and phospholipids. Moreover, nanoparticles and nanomicelles are widely used for efficient drug delivery with the aim of side effect reduction (Gogoi et al., 2018; Serati-Nouri et al., 2020; Nejati et al., 2021b). 

Nanoencapsulation of hydrophobic drugs represent various advantages in pharmaceutical sciences including controlled and stimuli-responsive drug release, long-term stability, non-toxicity, targeted drug delivery, and beneficial physiological properties (Amirsaadat et al., 2017; Patel et al., 2020). Among all the biomaterials, poly (lactic-co-glycolic acid) known as PLGA is an FDA-approved polymer extensively used in developing NPs due to high biocompatibility, small size, and biodegradability (Hossainzadeh et al., 2019; Norouzi et al., 2019; Samadzadeh et al., 2021). On the other hand, mucus permeability of PLGA is hindered according to hydrophobic interaction with mucin fibers (Mohammadian et al., 2016a; Mohammadian et al., 2016b; Adlravan et al., 2021). In this regard, copolymerization of PLGA with polyethylene glycol (PEG) or “PEGylation” improves cellular penetration and stability of PLGA in complex physiological environments (Dadashpour et al., 2017; Mousazadeh et al., 2020). Nanoencapsulation of hydrophobic components SIL with PLGA-PEG have improved the efficiency of drug delivery and reduced degradation rate in vivoThe aim of the present study was to evaluate anti-cancer and pro-apoptotic effects of SIL and SIL-NPs against T47D and MDA-MB231 breast cancer cells.

## Materials and Methods

*Materials*

 Glycolide, DL-Lactide, Dimethyl Sulfoxide (DMSO), Dichloromethane (DCM), Poly vinyl alcohol (PVA), Stannous 2-ethylhexanoate and Poly ethylene glycol was obtained from Merck chemical co. (Germany). Sil, were acquired from Sigma-Aldrich. Fetal bovine serum (FBS), Trypsin- EDTA, RPMI 1640, Penicillin G, Streptomycin, Trypsin-EDTA and TRIzol Reagent purchased from Gibco BRL. (Gaithersburg, MD, USA). First Strand cDNA synthesis Kit and SYBR Green PCR Master Mix were purchased from Fermentas (Vilnius, Lithuania).

*Methods*

*Copolymer synthesis*

PLGA-PEG copolymers were synthesized via ring-opening polymerization method under vacuum based on previous studies (Duan et al., 2007). For this purpose, lactide and glycolide monomers with the molar ratio of 4:1 in attendance of polyethylene glycol in the molten state were used. Lactide (2.882 g), glycolide (0.570 g), and (1.54 g) polyethylene glycol 46.5% w/w were heated to 140°C under nitrogen atmosphere in a bottleneck flask containing silicone oil to complete melting. After monomer melting, Sn (Oct)2 was added and the reaction mixture was further heated for 1 h, in order to complete polymerization under static vacuum. The synthesized copolymer was dissolved by dichloromethane followed by ice-cold diethyl ether precipitation. Lastly, the physicochemical properties of the product were evaluated by DLS and FE-SEM.

*Preparation of SIL-NPs*

SIL was encapsulated into PLGA-PEG copolymer through a slightly modified double emulsion method (W/O) (34). Briefly, 2 mg of SIL and 100 mg of pre-prepared PLGA-PEG copolymer (1:50) was dissolved in 5 ml of dichloromethane and 10 ml of 1% PVA (polyvinyl alcohol), respectively. The PLGA-PEG mixture was homogenized at 72,000 rpm for 20 minutes using a homogenizer and the PVA-SIL solution was gradually added to the mixture. After complete evaporation of the organic solvent (dichloromethane) by the rotary apparatus for 15 minutes at room temperature, the final w/o emulsion was centrifuged and purified by a 100 kDa cut off amicon filter at 15,000 rpm for 40 minutes to separate the encapsulated SIL. The DCM was completely vaporized using rotary evaporator under low vacuum. Then, two cycles of centrifugation were used to purification of NPs. Finally, the NPs formed washed twice with distilled water, lyophilized, and stored at 20^o^C for further experimentation.

*Physicochemical characterization of SIL-PLGA-PEG*

*Confirmation of SIL Loading by FTIR*

The identification of functional groups in the SIL, PLGA/PEG copolymer and SIL-NPs were analyzed using FTIR spectroscopy (PerkinElmer, Waltham, MA, USA) in the 4,000 cm^-1^ to 400cm^-1^ wavenumber range averaging 100 scans. 

*NPs morphology*

The shape, surface morphology and microstructure of prepared PLGA-PEG nanoparticles were studied by FE-SEM model Seron Technologies AIS 2100 (Korea). For this, a small quantity of samples was rinsed two times with purified water, freeze-dried, and coated with gold/palladium for scanning electron microscopic observation.

*SIL release profile from NPs*

Release of SIL from PLGA-PEG copolymer was assayed as follows: 25 mg of SIL-NPs was dissolved in 5 ml PBS at pH 4.4 and 7.4. Sample were set into a to dialysis membrane tubing (MW cut off: 3,000). The dialysis membrane tubing was introduced into vials containing 25 ml of buffer solution. At specific time, intervals from 0.5 h up to 120 h, SIL released samples were replaced with fresh PBS. Samples were centrifuged and supernatants were collected for UV-Visibility analysis at 570 nm (maximum absorption wavelength) using BEL LGS 53 spectrophotometer. The percentage of SIL release from NPs was measured by following equation: 

EE % = [(Drug Total - Drug Filtered) / (Drug Total)] × 100

*SIL encapsulation efficiency*

For the evaluation of encapsulation efficiency (EE), the freeze-dried drug encapsulated NPs were weighed and dissolved in H2O: CH3CN mixture (50/50, v/v) and then extracted with CH3OH. The resulted NPs were filtered using an Amicon 3 kDa centrifugal filter. Few microliters of filtrate were determined by high performance liquid chromatography (HPLC). The encapsulated drug is stated both as drug loading (DL) and entrapment efficiency (EE), characterized by Eqs. (1) and (2), respectively.

$\mathrm{Dl}\left(\%\right)=\frac{\mathrm{mass of drug in NPs}}{\mathrm{total NPs mass}}\times 100$ Eq. 1

EE%=mass of drug in NPstotal mass drug×100 Eq. 2

*Cell line maintenance*

Two human breast cancer cells, MCF-7 and MDA-MB-231 were cultured in RPMI-1640 media. The media was supplemented with 15% FBS, streptomycin (100 mg/mL), and penicillin (100 U/mL). Cells were incubated at 37°C with 15% CO_2_ in a humidified atmosphere. All experiments were carried out on logarithmically growing cells.

*Cell viability test*

In order to evaluate SIL and SIL-NPs effects on growth of cell, MTT assay was performed on MDA-MB231 and MCF-7 breast cancer cell lines. In this assay, 2 × 10^4^ cells/well were seeded in 96-well plates and cultured. Different concentrations (0-125 µM) of SIL and SIL-NPs were added after 24 h in equivalent doses for three-time intervals (24, 48, and 72 h). Control wells including cells without drug treatment and cells treated with free NPs were treated with an equivalent amount (1%) of ethanol. Following the treatment, the media containing SIL, SIL-NPs, and controls were eliminated closely and 200 μL of 2 mg/mL MTT dissolved in PBS was supplemented to each well, and the plates were coated with aluminum foil to incubate at 37°C for 4 hours with a 5% CO_2_ atmosphere. Thereafter, the contents of all each well were removed and 200 μl of DMSO mixed by pipette and left for 45 sec. Finally, the optical density (OD) was read with multi-well microplate reader (ELIZA reader, Organon Teknika).

*Real-time PCR assay*

RT-PCR assay was conducted to assess expression of *P53, caspase 3, 7, cyclin D1, survivin, Bax, Bcl2, *and *hTERT *genes under free and nanoformulated of SIL treatment. After 24 h drug exposure, cells were used for RNA extraction and complementary DNA (cDNA) synthesis using RNX-Plus and Prime Script RT reagent kits respectively. The pureness and concentration of total RNA were assessed by Thermo Scientific™ NanoDrop™ based on OD260/280 ratio measurements. cDNA synthesis was performed according to the protocol using 2μg of pure RNA as a template. Levels of *hTERT* gene expression was determined in a total volume of 14 mL per reaction using a Real-Time PCR Master Mix (BioFACT). Real-time PCR mixture contained SYBR Green qPCR Master Mix2x (7μL), a mixture of forward and reverse primers (0.3μL), diluted cDNA (1μL), and H_2_O PCR grade (4.7μL). The amplification condition was set as the following: preincubation for 15 min at 95°C, 45 cycles of denaturation for 10 s at 95°C, annealing for 30 s at 58°C and extension for 20 s at 72°C, which was followed by a melting curve analysis step. The efficiency of real-time PCR for targeted genes was relatively determined and normalized by the housekeeping *GAPDH* gene. The sequences of the forward and reverse primers are listed in Table 1. Each reaction was done in triplicate and the comparative Livak method (2^−ΔΔCT^) was employed for data analysis. 

*Statistical analysis*

Graph Pad Prism version 7.01 was used for statistical analyses of data. Data are presented as mean ± standard deviation and the difference between groups were determined by one-way analysis of variance (ANOVA). All experiments were performed in triplicate to assess reproducibility. P-values of p < 0.05 was considered as statistically significant.

## Results

*Characterization of SIL- NPs*

*SIL- NPs size, zeta potential, and morphology*

**Figure 1:**
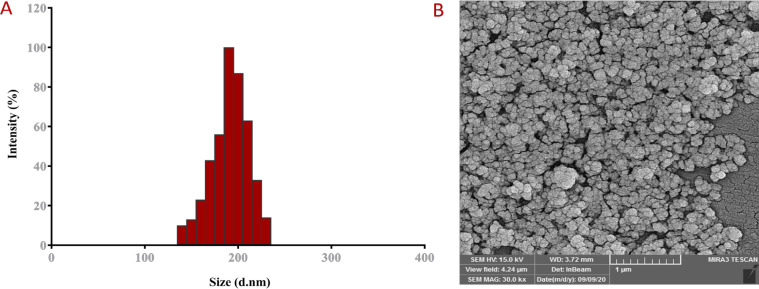
A DLS Histogram Showing the Size Distribution of SIL NPs. The average size ranged from 150–220 nm. B Field emission scanning electron microscopy (FE-SEM) image of surface morphology of SIL NPs

Table 2 summarized the size distribution, zeta potential, and polydispersity index of PLGA-PEG and SIL-NPs, measured by the DLS technique. PLGA-PEG can be emulsified in aqueous solution to form NPs. The size of PLGA-PEG copolymers must be small enough to prevent degradation by the reticuloendothelial system and persist in the circulatory system to be targeted appropriately. DLS analysis had revealed the average size of PLGA-PEG NPs equal to 220 nm with uniform dispersion while SIL-NPs represented an increased average size of 180 nm with a range between 150 and 220 nm indicating the SIL loading within PLGA-PEG core (Figure 1). Zeta potential analysis indicated information of the surface charge and high stability of the NPs. The appropriate surface charge of PLGA-PEG NPs permits their easy absorption through negatively charged cell membranes and which is contributed to efficient intracellular trafficking and would be useful for more application of PLGA-PEG in vitro studies. Encapsulation of SIL with PLGA-PEG has resulted in the increased surface charge of PLGA-PEG indicating the good stability of the SIL-PLGA-PEG NPs leading to better repulsion between the charged particles and prevents aggregation. SEM image revealed that the NPs possess homo-dispersed spherical morphology with a variable size in the range of 150+23.9 nm (Figure 1).

*FTIR studies*

**Figure 2:**
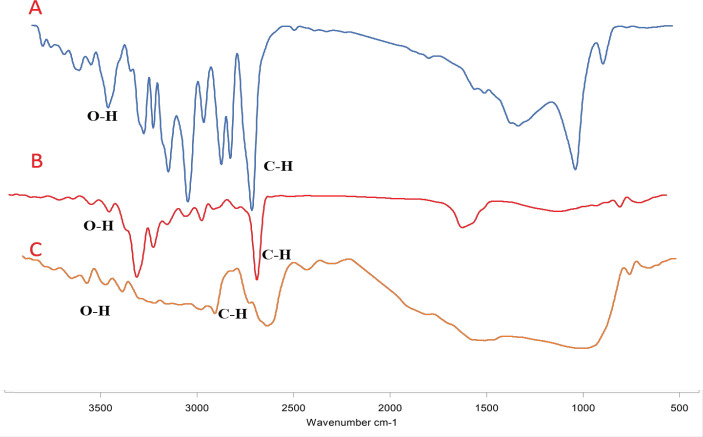
Infrared Spectra of SIL, PLGA/PEG and SIL-NPs

To further confirm SIL loading on designed NPs, FTIR spectroscopy was employed to illustrate the structure of PLGA-PEG NPs and SIL-loaded PLGA-PEG NPs (Figure 2). The FT-IR spectrum of free SIL represented an absorption band including 834 cm^-1^ related to C-O-C stretching, 1,277 cm^-1^ for aromatic C-O stretching, 1,464 and 1,512 cm^-1^ related to the skeleton vibration of aromatic C=C ring stretching, 1940 cm^-1^ demonstrating the C=O stretching, 2,854 to 2,925, and 3,431 cm^-1^ for C-H stretching. FT-IR spectrum of the shows the SIL-PLGA-PEG copolymers represented characteristic peaks at 2880.71 cm^-1^ and 1768.33 cm^-1^ which were related to the C-H and C=O bands stretching, respectively.

*In vitro drug release profile*

**Figure 3:**
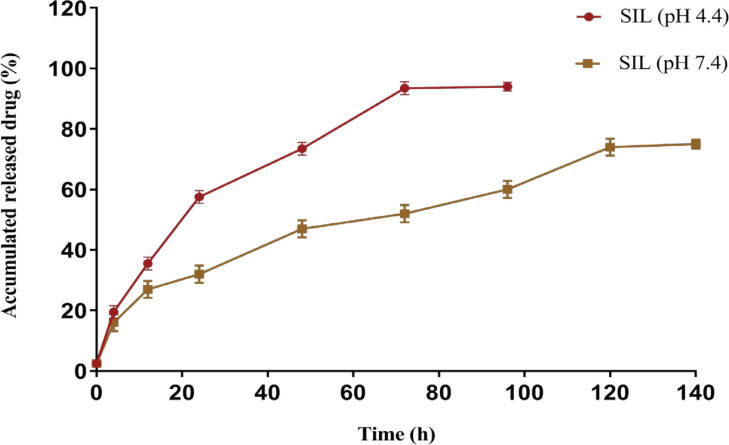
Cumulative Release (%) Behavior of SIL from NPs in Phosphate Buffered Saline (pH 7.4 and pH 4.4)

The release of SIL from PLGA-PEG nanoparticles was performed by dialysis against PBS at the physiological temperature of 37^o^C during a period of 168 hr. The release pattern of SIL from PLGA-PEG in pH= 7.4 and 4.4 is indicated in (Figure 3). Results demonstrated that a burst increase in SIL release occurs within 3 hours at a pH of 7.4, followed by a controlled release of SIL in 6 days. After 120 hr, approximately 75% of the SIL is released from the copolymer at pH = 7.4.

*SIL encapsulation efficiency*

The encapsulation efficiencies of SIL in the NPs were 82.32% at a copolymer/drug with a loading capacity of 11.1 ± 3.2%, respectively. Moreover, at higher concentrations of SIL, the DL and EE were initiated to be decreasing.

*Cytotoxicity studies*

**Figure 4:**
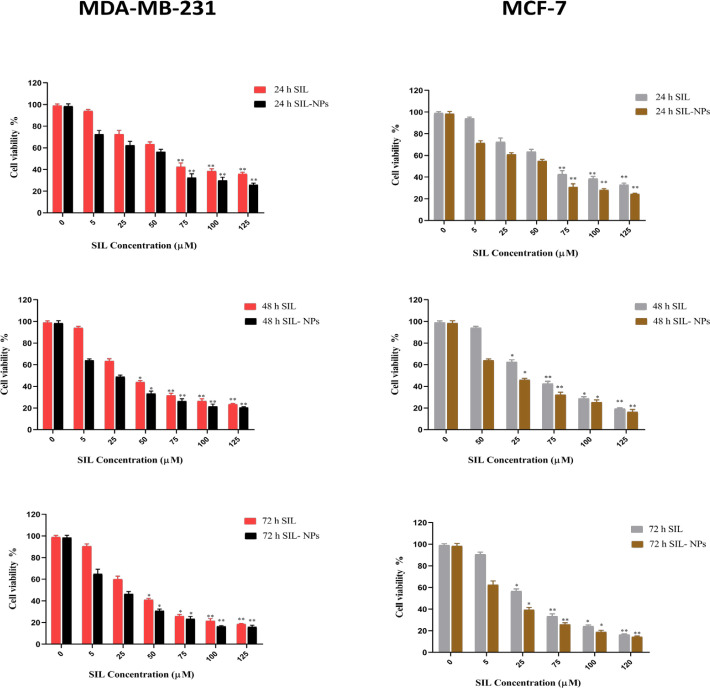
The In Vitro Cytotoxicity by MTT Assay. The viability of MDA-MB-231 and MCF-7 breast cancer cells receiving various treatments SIL and SIL-NPs. Error bars indicate standard deviations. (* P value < 0.05, ** P value < 0.001).

To evaluate the growth inhibition effect of SIL and SIL-NPs, MTT assay was applied using different doses (0-125µM) of free SIL and SIL-NPs on MCF-7 and MDA-MB231 breast cancer cells during 24, 48, and 72 h (Table 3). Cells without treatment were used as control groups. Table 2 shows the half maximal inhibitory concentration (IC_50_) values of SIL and SIL-NPs against MCF-7 and MDA-MB231 after treatment. Data analysis of MTT assay revealed the cytotoxicity effect of SIL in both free and nanocapsulated forms in a dose- and time- dependent manner. Regarding the diagrams of cytotoxicity analysis displayed in (Figure 4), SIL-NPs possess greater growth inhibitory effect than free SIL with a drastic reduction in SIL IC_50s_. In addition, the cytotoxicity of SIL-PLGA-PEG on MCF-7 cells was much more than on MDA-MB231. Treatment of both MDA-MB231 and MCF-7 with equal concentrations of SIL-NPs at certain times, have demonstrated that growth inhibitory effect of SIL-NPs represent a significant difference on MCF-7 compared with MDA-MB23. The results suggested that cytotoxicity of SIL-NPs is more effective in estrogen receptor positive breast cancer cells.

*Gene expression findings*

The expression of *P53, caspase 3, 7, cyclin D1, survivin, Bax, Bcl-2* and *hTERT* in MCF-7 and MDA-MB-231 cancer cell lines after exposure to SIL and SIL-NPs for 48 of incubation was determined by real-time PCR. As our results showed, caspase-3, caspase-7, P 53 and Bax (apoptosis markers) mRNA levels were meaningfully increased in the cells treated with nanoformulated forms of SIL compared to control. The decreased RNA transcription level of anti-apoptotic *hTERT, Cyclin D1, Survivin* and *Bcl-2* genes was observed in free and nanoformulated group compared with untreated cells. 

**Table 1:** Primer Sequences Used in Real-Time PCR Technique

Genes	Primer Sequence	PCR product size (bp)
*hTERT*	F: 5′-CCCATTTCATCAGCAAGTTTGG-3′	94
	R: 5′-CTTGGCTTTCAGGATGGAGTAG-3′	
*GAPDH*	F: 5′-CATGAGAAGTATGACAACAGCCT-3′	113
	R: 5′-AG TCCTTCCACGATACCAAAGT-3′	
*bcl-2*	F: 5′ GATGTGATGCCTCTGCGAAG -3′	93
	R: 5′-CATGCTGATGTCTCTGGAATCT-3′	
*Bax*	F: 5′-GGTTGTCGCCCTTTTCTA-3′	98
	R: 5′- CGGAGGAAGTCCAATGTC -3′	
*caspase-3*	F: 5′- TTCAGAGGGGATCGTTGTAGAAGTC -3′	178
	R: 5′- CAAGCTTGTCGGCATACTGTTTCAG -3′	
*caspase-7*	F: 5′-GGACCGAGTGCCCACTTATC-3′	149
	R: 5′-TCGCTTTGTCGAAGTTCTTGTT-3′	
*Cyclin D1*	F: 5′-AGACCTTCGTTGCCCTCTGT-3′	181
	R: 5′-CAGTCCGGGTCACACTTGAT-3′	
*Survivin*	F: 5′ GATGTGATGCCTCTGCGAAG -3′	103
	R: 5′-CATGCTGATGTCTCTGGAATCT-3′	
*P 53*	F: 5′- GACGGTGACACGCTTCCCTGGATT -3′	120
	R: 5′- GGGAACAAGAAGTGGAGAATGTCA -3′	

**Table 2:** Mean (±SD) Particle Diameter, Polydispersity (±SD) and Zeta Potential of Drug Loaded PLGA-PEG NPs

Formulation	Particle size (nm)a	Polydispersity index	Zeta potential (mV)a	Formulation	Particle size (nm)a	Polydispersity index
PLGA/PEG NPs	180±2.53	0.125	-5.48±0.13	PLGA/PEG NPs	180±2.54	0.125
SIL-loaded PLGA/PEG NPs	220±6.37	0.136	-6.8±0.26	SIL-loaded PLGA/PEG NPs	220±6.38	0.136

**Table 3:** IC_50_ Values for the Drug Formulations against MDA-MB-231and MCF-7 Breast Cancer Cell Lines after 24, 48, 72 h Incubation Time

time (h)	MDA-MB-231		MCF-7	
	SIL (µM)	SIL- NPs	SIL (µM)	SIL- NPs
24 h	65.11	55.23	53.68	48.21
48 h	55.29	48.35	47.41	43.51
72 h	45.11	39.54	27.14	19.13

**Figure 5:**
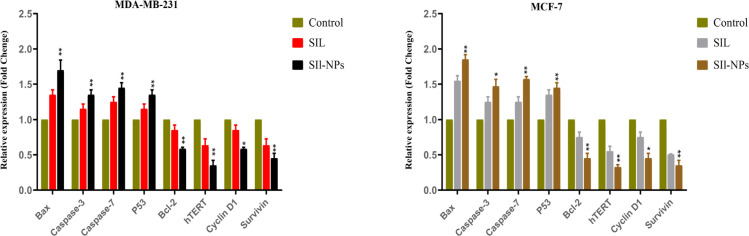
Relative mRNA Expression Levels of P53, Caspase 3, 7, Cyclin D1, Survivin and hTERT Genes in MDA-MB-231 and MCF-7 Breast Cancer Cell Line Treated with by Free and Nanoformulated form of Sil. *P < 0.05, **P < 0.01 and ***P < 0.001 vs. control was considered significant

## Discussion

The wide spectrum of cancer chemopreventive ability of SIL has been proved in several cases. SIL inhibits the proliferation and invasion of tumor cells in various cancers. It has been reported that SIL inhibits the invasion and proliferation of hepatocarcinoma cell lines via the induction of program cell death and cell cycle arrest (Boojar et al., 2016). Previous studies found that SIL inhibits ERK1/2 signaling, which indicates that the ERK1/2 signaling pathway may play as an upstream regulator that modulates the SIL-induced Bim signaling pathway. In addition, the effect of SIL on the prohibition of angiogenesis in hepatic cancers is associated with reduced secretion of vascular endothelial growth factor, CD34, and metalloproteinase-2 in a dose dependent manner (Li and Wang, 2016; Zappavigna et al., 2019). Likewise, high amounts of SIL in human colorectal mucosa was found to be deliverable through ingestion of nontoxic doses of SIL considered to be beneficial in early stages of colon tumorigenesis. SIL can potently hinder the proliferation of LoVo and HT-29 cells both in xenograft and vitro models through the induction of G1 and G2-M cell cycle arrest (Nafees et al., 2018). This effect is incorporated with lower levels of cell division cycle 25C (cdc25C), cyclins (A, B1, D1, D3, and E), and Cdc2/p34 as well as diminished activity of cyclin-dependent kinases (Errico et al., 2010)**.** SIL is also proved to have the ability to induce program cell death through activation of caspases 3 and 9 along (Zhang et al., 2018). Moreover, SIL treatment decreased the growth and invasiveness of renal cancer 786-O cell while restraining the growth of cell and inducing program cell death in cancer Caki-1 cells through prevention of EGF, ERK1/2 (Raina et al., 2016). In accordance with these findings, several studies have demonstrated the growth inhibitory effects SIL in rat models of urinary bladder cancer, associated with elevated levels of p53 expression, phosphorylation of ERK1/2 and phospho-p65, as well as downregulation of survivin, cyclin D1 (Barros et al., 2020). The use of Sil in breast cancer cells has caused cell apoptosis in MDA-MB-231 and MCF-7 cells, which synergized with inhibition of metastasis through several proteins including insulin growth factor receptor (Si et al., 2020). SIL decreased the expression of 12-O-tetradecanoylphorbol-13-acetate-induced MMP-9, EGFR ligand-induced CD44, and VEGF in a dose-dependent manner. Consistent with the results, SIL enhanced program cell death and stimulated G2-M cell cycle arrest in A2780/taxol cells associated with the downregulation of P-glycoproteins and survivin (Zheng et al., 2017). Synergistic Anti-proliferative ability of SIL in combination with other chemotherapeutics such as Doxorubicin, Metformin (MET), and Chrysin have been greatly assessed. Maasomi et.al, have evaluated the growth inhibitory effects of SIL and chrysin through regulation of hTERT and cyclin D1 in T47D breast cancer cells. Cell viability analysis have demonstrated that Chrysin or SIL can individually prevent the proliferation of T47D, and combination of these compounds resulted in synergistically growth inhibition (Maasomi et al., 2017). Chatran et al., (2018) have evaluated anticancer properties of SIL in mixture with MET on T47D cancer cells. In this study, gene expression analysis represented that in addition to individual drugs, co-delivery of SIL and MET synergistically decreased the cyclin D1 and hTERT expression levels. On the other hand, a plethora of investigations have focused on nanocapsulation of SIL in order to increase bioavailability and efficient delivery of this flavonoid. As a proof of concept, Amirsaadat et al., (2017) loaded SIL in magnetic PLGA-PEG-Fe3O4 NPs to evaluate the expression of hTERT and proliferation of A549 cancer cells. According to this investigation, in comparison with pure SIL, SIL loaded magnetic NPs reduced the hTERT expression more efficiently. Likewise, Amirsaadat et al., (2017) co-loaded SIL and MET on PLGA-PEG nanoparticles to evaluate its effect on the expression levels of apoptosis related and* hTERT* genes in T47D cancer cells. According to the results obtained, MET-SIL NPs demonstrated the more synergistic inhibitory effect on T47D cells growth. In the presented study, we aimed to evaluate cytotoxicity effect of SIL -NPs copolymer on MCF-7 and MDA-MB-231 breast cancer cell lines, compared based on estrogen receptor status. We assessed physicochemical properties of SIL-NPs as well as drug encapsulation efficiency and drug release profile. In addition, the effect of SIL and SIL-NPs on the expression of P53, caspase 3, 7, cyclin D1, survivin and hTERT was determined. In the current study, we synthesized PLGA-PEG with an average size of 220 nm based on the ring-opening melt polymerization method. SIL was encapsulated with an efficiency of about 83%. The chemical structure of SIL-PLGA-PEG was identified with FTIR and DLS analysis. DLS analysis confirmed that the NP was synthesized in the appropriate size. Furthermore, FE-SEM analysis revealed that SIL-PLGA-PEG possesses a much smaller particle size than those identified by DLS (180-250 nm). The profile of SIL release from PLGA-PEG was investigated by dialysis at 7.4 and 4.4. It was found that an instantaneous increase in release occurs within 3 hours at a pH of 7.4, which is followed by 6 days of controlled release of SIL resulted in about 88% of SIL release. The SIL released at pH 4.4, which is a simulation of the acidic state of lysosomes, occurs significantly faster than in pH 7.4 so that about 88% of the total SIL content was released within 6 days. The sustained drug discharge characteristic of PLGA-PEG lead to the fact that these NPs can be applied as an impressive vehicle for delivery of SIL. MTT assay represented that SIL and SIL-PLGA-PEG have cytotoxicity effects on both MCF-7 and MDA-MB231cell lines in a time- and dose-dependent manner. Real-time analysis has indicated that in comparison with pure SIL, nanocapsulated SIL represented a greater inhibitory effect on the expression of the* hTERT, Cyclin D1, Bcl-2* and *survivin* genes. Moreover, our results showed that SIL by upregulation of pro-apoptotic genes such as *caspase 3, caspase7* and* P53* induced apoptosis in cancerous cells. It appears that SIL-NPs can considerably trigger the downregulation and upregulation of apoptosis related and cell cycle control genes expression in breast cancer cell lines resulting in the inhibition of growth and proliferation which finally leads to cell death by apoptosis.

In conclusion, our findings revealed that PLGA-PEG NPs as a suitable and appropriate nanocarrier may preserve the drug and expose it inside the cells effectively. Moreover, SIL has strong anti-proliferative and anticancer effects with less side effects on MCf-7 and MDA-MB231 cell lines. According to this study, PLGA/PEG NPs prepared by w/o/w emulsion technique and Sil encapsulated into PLGA/PEG NPs to improves drug delivery system in order to develop novel and effective drug delivery systems to fight breast malignant tumor. The results have shown SIL in pure and nano form change and altered the expression level of P53, caspase 3, 7 and cyclin D1, survivin and hTERT. Particularly, the nano-coencapsulated state of SIl had a greater effect on the proapoptotic and antiapoptotic gnes. This preliminary study showed that the loading of the chemotheraputic molecules into PLGA/PEG might lead to develop a novel and safe nanodrug delivery systems for the effective treatment of breast cancer.

## Author Contribution Statement

The authors confirm contribution to the paper as follows: study conception and design: NZ, MD; data collection: AP,AM; analysis and interpretation of results: AF, MD; draft manuscript preparation: MD, AF, NZ. All authors reviewed the results and approved the final version of the manuscript.
